# Consumer heterogeneity for shared accommodations at pre-and-post adoption stages: Insights from travelers in Shanghai, China

**DOI:** 10.1371/journal.pone.0286868

**Published:** 2023-06-23

**Authors:** Lei Qin, Eddy S. Fang, Ivan Ka Wai Lai, Yu Han, Yidan Liu

**Affiliations:** 1 Business School, North Minzu University, Yinchuan, China; 2 International Business School Suzhou, Xi’an Jiaotong – Liverpool University, Suzhou, China; 3 Faculty of International Tourism and Management, City University of Macau, Macau, China; 4 School of Marketing and Logistics Management, Nanjing University of Finance and Economics, Nanjing, China; 5 Faculty of Business Economics, Shanghai Business School, Shanghai, China; Bialystok University of Technology: Politechnika Bialostocka, POLAND

## Abstract

As shared accommodation has become one of the most important market developments in the tourism industry, numerous contributions have emerged regarding travelers’ motivations to choose shared accommodation. A debated question, however, resides in the heterogeneity of travelers based on motivations. This paper aims to reconcile opposing perspectives by comparing motivation segmentation at two distinct phases of the adoption of this accommodation option: (i) before the first travel–potential users showing interest (n = 420) and (ii) after the first travel–current users (n = 420). Factor analysis, combined with clustering, is applied to both samples to identify underlying motivations and traveler segments. Interestingly, we find that factors defining choice motivations are relatively stable throughout the adoption process, but the heterogeneity of motivations among travelers is higher in users, increasing from three to six clusters. This suggests that travelers’ motivations are dynamic and dependent on the phase of adoption.

## Introduction

Shared accommodations refer to the owned or rented properties that hosts are willing to share with travelers in the short term, such as Airbnb (https://www.airbnb.cn), Xiaozhu (https://www.xiaozhu.com), Tujia (https://m.fvt.tujia.com), etc. [1]. Travelers have their own bedrooms and share living rooms, bathrooms, kitchens, etc. [2]. Compared with hotels that exist strong institutional specifications, shared accommodations are characterized by higher trust and lower social distance between hosts and customers [3]. The global shared accommodation booking amount increased by 25%, increased to 103.7 million from April to June 2022, compared to 2021 with a 24% increase from 2019 before the COVID pandemic [1]. The Chinese shared accommodation revenue accounted for 5.9% of the tourism accommodation market in 2021, rising from 4.4% in 2017 to 5.9% in 2021 [4]. The overall increase of 1.5% indicates that the share of shared accommodation in China’s accommodation industry has increased, and in the future, shared accommodations can be the main accommodation mode for Chinese young travelers. Therefore, it is crucial to understand Chinese travelers’ motivations to engage in shared accommodations and distinguish new and repeat users’ motivations [5].

Previous studies have considered homogenous motivations and concluded that travelers’ rationale for adopting shared accommodations often finds roots in the cheaper price relative to the space rented [6], or the new type of travel experience [5]. The scale of shared accommodation housing is generally relatively small and thus each has its own characteristics. Therefore, different types of travelers will choose different types of shared accommodations. With more and more shared accommodation houses, the competition is getting fiercer, so it is crucial to find out who uses shared properties and the underlying reasons to make the marketing strategies effective [7, 8]. Only in this way, travelers can be placed in the proper market segment, which enables shared accommodations to make effective marketing strategies.

Within existent tourism and hospitality studies, scholars segmented travelers by using demographic factors (e.g., gender, age, education background, financial status, etc.) or motivational factors (e.g., lower price, home feeling, better location, better household facilities, etc.) [5, 9]. The tourism market segmentation studies aimed to divide travelers into groups with internal homogeneity but great heterogeneity with the rest of the groups by assuming that the overall travelers are heterogeneous based on motivations [10]. However, existing studies only took a significant look at heterogeneous issues for travelers who have already used tourism services or accommodations [5, 11, 12], with limited consideration of the pre-trip stage of shared accommodation adoptions (e.g., potential travelers who have never adopted shared accommodations).

Shared accommodation is viewed as an innovation for its effective and novel combination of existing resources, which leads to the introduction and expansion of the structures of the tourism market and industry [13]. Most of the previous findings that have centred on motivational differences among travelers at different adoption stages of shared accommodations can be supported by Rogers’ [14] Innovation Adoption Theory, which summarised five categories of adopters with diverse characteristics in using innovative technology, services or products: a) innovators, b) early adopters, c) early majority, d) late majority, and e) laggards. The main purpose of this study is to analyse the heterogeneity in different stages of shared accommodation adoption, which mainly addresses two types of travelers: pre-adopters (specifically referring to potential travelers) and post-adopters. Travelers who are unaware of existing tourism service or products, travelers who are aware of them but will not use them and retractors who tend to have a motivation are not addressed in this study.

There is a need to have a study to classify the shared accommodation segments based on potential customers and experienced customers. To have consistent marketing strategies, shared accommodation marketers should know the changes in traveler segments from the pre-trip stage to the post-trip stage. Knowing the traveler segments based on the pre-trip stage can help shared accommodation marketers to design their advertising strategies to create potential customers’ awareness and interest. Understanding the traveler segments based on the post-trip stage can help shared accommodations refine their market positions and strengthen existing customers’ repurchasing intentions.

Interestingly, the findings of this study reveal that the heterogeneity of motivations among travelers is higher in post-adoption, increasing from three to six clusters. Travelers’ clusters increase and become distinct motivation-based groups after adoption. Instead of invalidating specific assumptions used in the literature in terms of motivation heterogeneity, this paper reconciles existent findings by sorting out and connecting them in the adoption process. A better understanding of travelers’ motivations for using shared accommodations, the segments identified and profiled, and the motivation evolution from travelers at pre-adoption to the post-adoption stage are illustrated.

Motivations for pre-adopters and post-adopters (refer to actual users) were discussed in previous studies. Pre-adopters usually seek shared properties with lower prices [15]. In comparison, post-adopters tend to be clearer on the benefits of shared properties in functional and experiential aspects, thus they would make reservations based on preferences [5]. However, tourist segmentation studies mainly focused on users [5, 8, 16]. From this point of view, this study contributes to highlighting the importance of studying pre-adopters’ (refer to potential users, non-users who have no motivations were excluded in this study) segmentations, whose market strategies needed to be distinguished from post-adopters. Moreover, how travelers’ motivations evolve before and after travel was not fully investigated in existing studies. This study, therefore, contributes to enriching our knowledge of how the tourism market evolves from homogeneity to heterogeneity over time, by using shared accommodation as a case study. Besides, this research contributes methodologically to market segmentation research in the tourism context by providing an innovative methodology for comparing tourist segments at different adoption phases. Last, this study helps provide overall marketing insights, to not only encourage post-adopters’ engagement, but also to attract potential users of shared accommodations to have a first attempt.

This paper is structured as follows: Section 2 discusses the previous literature on the topic of travelers’ innovation adoption types, their motivations and market segmentations. Section 3 describes the methodology used in this paper, while Section 4 lays down the results of the factor analysis and clustering. Section 5 discusses the findings and draws implications and limitations.

## Literature review

### Innovation adopter types

The Innovation adoption theory (IAT) is capable of providing a rationale to categorize travelers at a number of adoption stages in adopting a tourism service or product, from the pre-adoption stage (acquire initial knowledge, produce an attitude, and decide to adopt or not) to the post-adoption stage (seek to strengthen decisions on adoption decisions or retract) [14], which helps to divide travelers into pre-and-post adopters. The current study, therefore, divides travelers into pre-adopters (potential users who are aware of this innovation and will use it) and post-adopters (current users who have adopted this innovation).

Besides, the IAT can provide a useful perspective to extract and understand motivations because consumers’ innovation adoption can shape their motivations [14]. De Larrea et al. [17] illustrated that the beliefs that users hold for repeat usage are not likely to be the same beliefs that yield the initial adoption. Depending on the users’ experience, different factors can exert different effects on the intention and use of innovative services or products [18]. Besides, knowledge derived from past behavior can shape the users’ behavioral intentions [19]. Previous literature has indeed linked individuals at different innovation adoption processes with motivational differences [5]. Guttentag et al. [5] indicate that Airbnb users are more strongly motivated by practical motivations than experiential motivations. Maghrifani et al. [20] reveal that repeat travelers have stronger interaction-seeking motivations while potential travelers have stronger novelty- and assurance-seeking motivations. The current study, therefore, explores the motivations for using shared accommodations—fits with the IAT that illustrates potential and current users’ different motivations for product choices.

### Motivations of using shared accommodations

Motivation is a term that explains the reason why individuals prefer to participate in a particular activity [8]. With the rapid development of shared accommodations in the tourism industry, contributions have emerged to travelers’ motivations to use shared properties [2, 5]. Researchers have tried to use economic, social, functional, and experiential motivations to explain the variations between potential customers and experienced customers [5, 19].

For potential customers, shared accommodations not only save money (refer to economic motivations), but also provide an opportunity to gain shared accommodation ethos and philosophy, which enable travelers to perceive an added value of this industry [21]. Besides, potential customers are more willing to accept others’ recommendations (refer to social motivations generated by learning and experiencing the social environment based on individuals’ social and cultural needs) to save time and make a better selection [22], which is likely to yield tourism market homogeneity in travelers’ preferences.

Experienced customers tend to be more confident in booking shared rooms according to their prior knowledge of both experiential and functional aspects formed by their actual experience of using it [19]. Specifically, experiential motivations (e.g., novelty experiences, interactions, shared accommodation ethos, etc.) are treated as the major motivating force for transactions in the sharing economy [23]. Functional motivations (e.g., better location, better household facilities, larger space, etc.) play a role in enhancing the service performance level [24].

In this sense, potential customers are easily motivated by social and economic motivations, but experienced customers are attracted by functional and experiential motivations. It may infer that those social and economic motivations group the homogeneity of the tourism market and functional and experiential motivations divide the heterogeneity of the tourism market [5].

#### Tourism marketing segmentation research

Motivation-based segmentation refers to the market that could be categorized into various groups with internal similarities based on motivations [5, 8]. Motivations are widely used for the segmentation of tourism marketing [8, 25, 26]. Chung et al., [25] chose to segment independent business guests of luxury Seoul hotels by using benefits. Wen and Huang [8] segmented Chinese travelers visiting Israel by integrating cultural values into motivation-based segmentation. Khoo-Lattimore and Prayag [26] segmented “girlfriend getaway” travelers according to their preferences for accommodation options.

In the Chinese tourism market, there are diversified Chinese travelers based on their behavior practices and motivations [9]. According to Sparks and Pan [27], autonomy is more valued by younger Chinese travelers. Yu [16] analyzed the reason for Chinese tourists choosing Airbnb and indicated the segmentation of Chinese tourists in motivations, including Easy-going consumers, Non-collaborative consumers, Egocentric consumers and Captious consumers. However, there is currently limited research focusing on the explorations of potential and actual users of shared accommodations. Potential users are usually motivated to use shared accommodations for similar reasons, including lower prices and friend recommendations [28]. However, there are various shared rooms with unique characteristics, and their potential appeals include economic, functional, social connections, and experiential benefits that may not be obtained simultaneously, thus segmentation is appropriate for the shared accommodation market [5]. As we know, travelers are generally different in potentially noteworthy ways, such as age, and emotional connections to shared accommodations.

These studies have provided valuable insights into travelers’ motivations. However, these studies didn’t fully understand the pre-trip market segments, which play a crucial role in developing the shared accommodation market the lack of full consideration of this group of travelers tends to yield imperfectly effective marketing strategies [16]. In addition, academic and tourism industry stakeholders also want to know how those groups of tourists change their motivations from the pre-trip stage to the post-trip stage. Knowing how motivations evolve from pre-to-post adopters enables the shared accommodation operators to have overall marketing insights, to relocate their resources to improve their services and marketers to set up customer relationship strategies to attract potential customers and retain existing guests.

## Methodology

### Research design and data analysis

This research studies the motivations for shared accommodations with data collected from two questionnaires distributed to pre-adopters and post-adopters respectively. Particularly, questionnaires were designed in English to keep the originality of measurements, followed by back translating the questionnaires into Chinese by two academic scholars [29]. This study was approved by Xi’an Jiaotong-Liverpool University ethics committee and all respondents were provided informed consent on the first page of the online questionnaire. A pilot data collection was completed in April 2018. The questionnaires were first developed based on the existing studies and a few adjustments were made as some further insights were obtained from six pilot interviews (three pre-adopter and three post-adopters). The interview questions consisted of three sections: a) introductory questions, e.g., introduce yourself and this trip; b) motivation questions, including a variety of motivations c) demographic questions, including age, gender, educational background, etc.

SPSS.20 is used for conducting factor analysis, cluster analysis and discriminant analysis [5]. First, descriptive analysis is used to conduct an overview of travelers’ responses to survey questions. Second, an exploratory factor analysis by using the direct oblimin oblique rotation and principal axis factoring extraction is conducted for the 22 motivations, in order to identify the main motivation factors. Third, for remaining some meaningful variance that is lost from the factor analysis [30], the cluster analysis is used to aggregate the diverse groups of travelers based on 22 motivational items rated on a 6-level Likert scale (1 = strongly disagree, 6 = strongly agree), for the reason that even number Likert scale is capable of obtaining clearer attitudes or behaviors of respondents rather than ambiguous options [5, 26]. This study conducts the hierarchical cluster analysis to determine the optimal number of clusters by observing the significant change points via the agglomeration coefficients tables, followed by conducting a k-means cluster analysis to segment motivation-based travelers by inputting the optimal cluster number [5]. Last, a discriminant analysis was employed to confirm the validity of cluster solutions.

### Questionnaire design

One questionnaire for post-adopters and one questionnaire for pre-adopters were distributed online respectively. A screening question of “Have you ever used shared accommodations?” was used to distribute two different questionnaires to respondents. Each questionnaire is composed of two parts: Section 1 contains 22 items based on motivations for shared accommodations and Section 2 includes demographic questions. The motivation questions were the same as those while demographic questions were slightly different for pre-and-post adopters. Post-adopters were required to fill in the questionnaire according to their recent shared accommodation experience in the past 12 months and pre-adopters needed to answer the questions based on their expectations of shared accommodations in the coming 12 months.

In this exploratory research, the motivational scale items were organized with a particular concern for the research objectives, which were primarily based on previously observed motivations in the existing shared accommodation literature [2, 3]. The 22-item Likert questions (See S1 Table) were rooted in the comparison of the traveler heterogeneity between pre-adoption and post-adoption stages.

### Data collection

The data collection focused on travelers living in the city of Shanghai, China. The rationale is as follows: firstly, the traffic in Shanghai is convenient, including numerous trains going to a number of domestic cities and two international airports that enable travelers to fly across the world; secondly, this new accommodation mode has spread quickly in Shanghai, therefore, travelers living in Shanghai could be more active and tend to engage in shared properties by having numerous opportunities to acquire shared economy philosophy and ethos as the first-tier city [1].

Respondents were recruited to complete an online survey via wjx.cn (the largest online panel data service in China, akin to Qualtrics) from December 2018 to March 2019 and these respondents could receive compensation for their responses from wjx.cn. The data collection service of wjx.cn includes providing reliable data and recruiting respondents under a number of criteria. Before recruiting particular respondents, researchers could set specific criteria of equal-sized pre-and-post adopters of shared accommodations, then wjx.cn would contact respondents via emails in their large-scale respondents’ database until a specified number of questionnaires were collected.

A total number of 904 responses were collected for this study, 840 of them were valid responses after incomplete questionnaires were discarded (92.9%). Among the invalid responses, 13 were from post-adopters and 51 were from pre-adopters. Consequently, valid responses were received from the purposive sampling approach with a quota frame based on the equal distribution of pre-adopters (who intended to first use shared accommodations in the next 12 months) and post-adopters (who had used shared properties during the previous 12 months), including 420 respondents (50%) of each sub-sample respectively. The underlying reasons for conducting the purposive sampling with equal-sized groups tend to yield a more reliable result of comparison that can evaluate motivations for each group and look at the overall travelers’ conditions [31].

## Results

### Descriptive statistics for pre-and-post adopters’ motivations

Pre-adopters and post-adopters display similar characteristics (See S2 Table), in this context, the majority of respondents are female and in younger age groups, which matches the situation of the shared accommodation industry. Shanghai is one of the largest tourist sources in China because of Shanghai residents’ strong consumption ability and high-income level. The number of shared accommodation orders of Shanghai residents during the May Day holiday in 2023 reached 5.17 times that of the May Day holiday in 2019, indicating a strong recovery in the tourism market. Moreover, Shanghai residents have the highest consumption of shared accommodations, with an average overnight price of nearly 850 yuan, dominated by the generation after the 90s and 00s that mainly consisted of new users, accounting for over 72% of the shared accommodation consumption [32].

Table 1 reveals that pre-adopters are more likely to seek loadings with cheaper prices and more distinctive shared accommodation characteristics, with higher mean of E1 (4.133), E2 (4.088), E3 (4.002) and E4 (3.338) than post-adopters. The desire for shared accommodation ethos was included in the perceived value of shared properties, which can be distinguished from traditional hotels [7]. Moreover, social connections related to travel parties and word-of-mouth communication motivated pre-adopters more than post-adopters, with a greater mean of S1 (3.093), S2 (3.769) and S4 (4.233) than post-adopters. A possible explanation is that potential users without shared accommodation experiences are more likely influenced by others’ suggestions and recommendations. In addition, much attention is paid to the role of hosts that share local tips and travel suggestions with shared accommodation users [21]. The agreement on functional attributes is stronger in post-adopters because they are clearer in which kind of shared properties they prefer and which functional attributes dominated more in their selections, with greater mean of F1 (4.817), F3 (4.669), F4 (4.193), F5 (3.979), F6 (4.541) than pre-adopters. Last, the agreement on experiential benefits is stronger in post-adopters for they have had actual experiences in using shared properties, with higher mean of Ex2 (4.405), Ex3 (4.610), Ex4 (4.895), Ex5 (4.712), Ex6 (4.712), Ex7 (4.348) and Ex8 (4.893). Compared with potential users who answered the experiential questions based on their expectations of shared accommodations, the past experience of using shared accommodations tends to be helpful for them in segmenting shared loadings and selecting their ideal rooms more easily.

**Table 1:** Descriptive statistics for pre-and-post adopters’ motivations.

		Pre-adopters (N = 420)		Post-adopters (N = 420)	
		Mean	SD	Mean	SD
	**Economic and perceived value**				
E1	1. Cheaper price	4.133	1.322	3.969	1.304
E2	12. Live in a non-touristy	4.088	1.462	3.810	1.479
E3	17. It is environmentally friendly	4.002	1.402	3.748	1.353
E4	18. Shared accommodation philosophy	3.338	1.483	3.121	1.423
	**Social connections**				
S1	5. The others want to live in it	3.093	1.463	2.910	1.280
S2	6. Others recommend it to me	3.769	1.367	3.712	1.320
S3	8. Consider travel party size	4.074	1.422	4.202	1.399
S4	10. Share my experiences with my friends or relatives	4.233	1.340	4.079	1.358
	**Functional Attributes**				
F1	2. Better location	4.736	1.131	4.817	0.985
F2	3. Larger space	4.333	1.243	4.264	1.137
F3	4. Better household facilities	4.631	1.216	4.669	1.140
F4	7. Get useful travel tips and information from my host	4.107	1.302	4.193	1.323
F5	9. Additional Services	3.900	1.369	3.979	1.342
F6	13. More accommodation options	4.505	1.315	4.541	1.236
	**Experiential benefits**				
Ex1	11. Nonstandard experience	4.624	1.229	4.536	1.328
Ex2	14. Interact with the host and the locals	4.393	1.347	4.405	1.357
Ex3	15. Home-feeling	4.588	1.327	4.610	1.261
Ex4	16. New and different accommodation mode	4.886	1.125	4.895	1.092
Ex5	19. Have an authentic local experience	4.655	1.192	4.712	1.193
Ex6	20. Have fun	4.693	1.172	4.712	1.142
Ex7	21. I enjoy staying in it	4.302	1.256	4.348	1.299
Ex8	22. The new accommodation will make me happy	4.783	1.076	4.893	1.050

^a^SD represents the standard deviation.

### Cluster analysis results

In the present study, the multicollinearity problem was first assessed to ensure that no variables had correlations over 0.9 [33]. The two-stage cluster analysis was used widely in tourism and hospitality studies, a hierarchical cluster analysis was first conducted to obtain the optimal cluster number, followed by a k-means cluster analysis applied to effectively group respondents [5]. Moreover, the changes within clusters were examined subsequently [30]. The 22 motivations, including the removed five motivations in factor analysis, were inserted in cluster analysis. The rationale is that some meaningful variance is lost from the factor analysis, which means that factor-cluster analysis cannot reflect the original data of respondents when compressing variables into factors [30]. To make the interpretation easier, the original 22 factors along with the factor analysis result are used to explain the results of the cluster analysis.

For pre-adopters, the agglomeration coefficient obtained from the hierarchical cluster approach indicates that the most significant increase of agglomeration coefficient is from stage 418 to 417 (n = 420), presenting that the optimal cluster number is three. As shown in Table 2, a three-solution cluster emerges. The first cluster (n = 142, 33.8%), labelled “Recommendation Seekers”, holds less obvious motivations while this group of travelers are relatively easily attracted to use shared properties by recommendations of friends or travel companions and cheaper accommodation costs. The second cluster (n = 122, 29.0%) is termed “Experiential seekers”, as they hold the most favorable motivations for experiential benefits. Travelers from the last cluster (n = 156, 37.2%) reveal a rather favorable motivation for practical benefits, including lower prices and other functional benefits, thus they are termed “Practical seekers”.

**Table 2:** Cluster analysis results for pre-adopters.

	Pre-adopters				
	Recommendation Seekers	Experiential Seekers	Practical Seekers	F-value	Sig.
**Economic and perceived value**					
E1	4.09	3.87	4.38	5.293	0.005
E2	3.01	4.65	4.63	81.049	0
E3	3.32	4.02	4.60	36.152	0
E4	2.73	3.01	4.15	46.308	0
**Social Connection**					
S1	2.90	1.89	4.21	151.985	0
S2	3.65	2.87	4.58	72.796	0
S3	3.94	3.33	4.78	44.456	0
**Functional Benefits**					
F1	4.35	4.83	5.02	14.685	0
F2	3.77	4.31	4.87	33.524	0
F3	4.01	4.83	5.04	32.990	0
F4	3.62	4.08	4.57	21.830	0
F5	3.49	3.54	4.56	33.063	0
F6	3.97	4.54	4.96	23.358	0
**Experiential Benefits**					
Ex1	3.53	4.20	4.90	47.403	0
Ex2	3.94	5.01	4.95	39.874	0
Ex3	3.63	4.72	4.83	40.784	0
Ex4	3.68	5.05	5.06	66.528	0
Ex5	4.30	5.31	5.08	35.107	0
Ex6	3.94	5.09	4.97	48.175	0
Ex7	4.10	4.94	5.04	31.920	0
Ex8	3.59	4.49	4.80	43.927	0
Ex9	4.25	5.16	4.98	31.633	0
**Number in clusters**	142	122	156		
**% of Sample**	33.80%	29.00%	37.20%		

^a^All factors were measured by a six-scaled Likert questions ranged from 1 (strongly disagree) to 6 (strongly agree).

^b^The cluster mean scores represent their deviation from the sample mean.

Pre-adopters are more homogeneous because they are only aware of shared accommodations and are interested in using this tourism accommodation option. However, their knowledge of how to use shared accommodations properly (e.g., competencies of booking shared properties, preferences of shared properties and competencies of interaction with the hosts), their perceptions of shared accommodation attributes and their understanding of the shared accommodation industry are very limited [34]. Moreover, pre-adopters’ needs and desires are homogenized so that travelers were expected to prefer standard services or products of high quality and low price as compared to more customized high price services or products [35]. The rationale is that pre-adopters’ knowledge acquirement still stays at the initial level (awareness of knowledge), which restricts their perceptions of shared accommodation attributes. The limited knowledge of shared accommodation attributes makes it less possible for them to know their own preferences in selecting shared properties among a variety of loading listings [7]. After travelers have adopted the innovation service, their understanding of how to use the innovation properly and how the innovation works can be developed [14].

For post-adopters, the Agglomeration Schedule indicates that the most significant increase in agglomeration coefficient is from stage 415 to 414 (n = 420), which suggests that the optimal cluster number is six. Based on travelers’ motivations to select shared accommodations, the six clusters can be named Ethos travelers, Interactive travelers, Recommendation Seekers, Money savers, Novelty & Happiness seekers and Residual travelers (See Table 3). Particularly, Ethos travelers were strongly motivated by shared accommodation ethos, which means that they have developed a deeper understanding and general acceptance of shared accommodations and have already realized its practical and experiential benefits. According to the significant strong agreement in interaction benefits, social connections, cheaper price and novelty & enjoyment respectively, travelers are segmented as Interactive Travelers, Recommendation Seekers, Money Savers and Novelty & Happiness Seekers. The sixth cluster, Residual Travelers, is the remained group with the smallest number of travelers. They were less motivated by shared accommodation ethos, which yields less agreement in other motivations because of the limited interest in shared accommodation philosophy.

**Table 3:** Cluster analysis results for post-adopters.

	Post-adopters							
	Ethos Travelers	Interactive Travelers	Recommendation Seekers	Money Savers	Novelty & Happiness Seekers	Residual Travelers	F-value	Sig.
**Economic and perceived value**								
E1	4.30	3.41	4.24	4.33	3.66	3.77	7.786	0
E2	4.53	3.89	3.73	3.37	3.55	2.85	11.028	0
E3	4.97	3.31	4.23	2.65	3.38	2.87	54.213	0
E4	4.44	2.82	3.09	2.02	3.00	2.31	43.921	0
**Social Connection**								
S1	3.56	2.20	3.21	3.24	2.68	2.10	20.165	0
S2	4.56	2.98	4.21	3.87	3.26	2.62	31.973	0
S3	4.88	4.36	4.46	4.81	2.19	2.97	54.895	0
**Functional Benefits**								
F1	5.15	4.77	4.66	4.92	4.85	4.10	7.657	0
F2	5.04	4.26	3.96	4.24	3.87	3.26	22.653	0
F3	5.10	4.97	4.07	4.70	4.57	3.90	13.675	0
F4	5.03	4.53	4.60	3.41	3.30	2.72	44.606	0
F5	5.00	4.14	4.13	3.43	2.98	2.67	40.190	0
F6	5.13	4.60	4.23	4.17	4.60	3.87	10.182	0
**Experiential Benefits**								
Ex1	4.95	4.47	4.40	2.97	3.45	2.74	43.980	0
Ex2	5.24	4.93	4.16	3.86	5.02	2.87	36.191	
Ex3	5.15	5.18	4.31	3.32	3.96	2.95	48.023	0
Ex4	5.20	5.26	4.11	3.90	4.64	3.41	31.108	0
Ex5	5.26	5.22	4.20	4.92	5.04	4.15	15.825	0
Ex6	5.37	5.06	4.36	3.60	5.13	4.00	33.472	0
Ex7	5.18	5.02	4.13	4.46	5.19	3.56	23.882	0
Ex8	4.94	4.55	3.69	4.25	5.04	2.74	31.627	0
Ex9	5.34	5.23	4.37	4.83	5.06	3.69	25.562	0
**Number in clusters**	105	96	70	63	47	39		
**% of Sample**	25.00%	22.80%	16.70%	15.00%	11.20%	9.30%		

^a^All factors were measured by a six-scaled Likert questions ranged from 1 (strongly disagree) to 6 (strongly agree).

^b^The cluster mean scores represent their deviation from the sample mean.

Significant differences between travelers at the pre-adoption and post-adoption stages are found by comparing pre-adopters and post-adopters. Travelers’ cluster becomes plural and different after adoption, increasing from three to six clusters. Compared with pre-adopters, post-adopters reveal higher knowledge acquirement of shared accommodations, as they have access to three types of knowledge influencing the innovation adoption decisions. Before the actual involvement in shared accommodations, travelers’ knowledge significantly influences their decision-making. With an awareness-knowledge of innovation, travelers would make their decisions, represent their initial exposure to innovation and understand how innovation works [14]. In this regard, pre-adopters’ motivations are more homogenous, because they prefer standard service or products of high quality and low price, as compared to more customized high-price services or products [35], for they still stay at the awareness level of innovation knowledge. Compared with pre-adopters, post-adopters’ perceptions of attributes of shared accommodations, along with their preferences for different types of shared properties, emerge gradually as their growth experiences of shared accommodations. Pre-adopters who hold less experiential and functional knowledge of shared accommodations tend to rely more on recommendations of friends or travel companions. Therefore, post-adopters are more heterogeneous in motivation-based segmentation because they prefer different attributes of shared accommodation and their in-depth understanding of the tourism accommodation mode after experiencing the benefits of shared properties. However, pre-adopters are more homogenous because they are more likely to adopt shared properties with higher quality and lower prices [15].

A better understanding and differentiation among segments of post-adopters is crucial to promote travelers’ repeat participation. Six segments of post-adopters differ significantly in various motivations (See Table 4). In terms of personal demographics, six clusters are different in age, job and household composition at 1% significance level. Furthermore, with regard to travel characteristics and shared accommodation usage profiles, the six segments differ significantly in sources and types of shared accommodations, but not in travel purposes. A possible explanation is that the travel purpose of the majority of travelers is leisure (94.0%) rather than business or VRF (visiting relatives and friends).

**Table 4:** Profiles of post-adopters’ clusters.

	Ethos Travelers	Interactive Travelers	Recommendation Seekers	Money Savers	Novelty & Happiness Seekers	Residual Travelers	Average	Chi-Square	P-value
**Gender**								8.760	.119
Male	35.2%	29.2%	32.9%	31.7%	23.4%	51.3%	33.1%		
Female	64.8%	70.8%	67.1%	68.3%	76.6%	48.7%	66.9%		
**Age**								21.045***	.003
≤30	50.5%	63.5%	64.3%	69.8%	63.8%	87.2%	63.6%		
>30	49.5%	36.5%	35.7%	30.2%	36.4%	12.8%	36.4%		
**Job**								24.563***	.006
Company staff	54.3%	46.9%	41.4%	28.6%	48.9%	28.2%	43.6%		
Student	14.3%	20.8%	24.3%	41.3%	21.3%	38.5%	24.5%		
Others (financial etc.)	31.4%	32.3%	34.3%	30.2%	29.8%	33.3%	31.9%		
**Education**								13.684**	.018
College and below	31.4%	18.8%	12.9%	15.9%	21.3%	33.3%	22.1%		
Bachelor and above	68.6%	81.2%	87.1%	84.1%	78.7%	66.7%	77.9%		
**Financial status**								7.819	.167
Below average	25.7%	20.8%	28.6%	28.6%	31.9%	43.6%	27.9%		
Above average	74.3%	79.2%	71.4%	71.4%	68.1%	56.4%	72.1%		
**Travel purpose**								2.078	.838
Leisure	92.4%	95.8%	92.9%	93.7%	93.6%	97.4%	94.0%		
Others (VFR, business, etc.)	7.6%	4.2%	7.1%	6.3%	6.4%	2.6%	6.0%		
**Where did you hear SA?**								23.969***	.000
Recommend by friends, etc.	29.8%	9.4%	22.9%	17.5%	23.3%	23.1%	23.3%		
Web and apps	70.2%	90.6%	77.1%	82.5%	76.7%	62.9%	76.7%		
**Number of other travelers**								21.905**	.016
0–1	11.4%	13.5%	15.7%	22.2%	34.0%	28.2%	18.3%		
2–3	55.2%	54.2%	61.4%	42.9%	51.1%	46.2%	52.9%		
≥4	33.4%	32.3%	22.9%	34.9%	14.9%	25.6%	28.8%		
**Travel composition**								9.832	.455
Friends	48.6%	54.2%	52.9%	55.6%	51.1%	61.5%	53.1%		
Alone/relatives	21.0%	13.5%	21.4%	15.9%	8.5%	7.7%	16.0%		
Spouse/children	30.5%	32.3%	25.7%	28.6%	40.4%	30.8%	30.9%		
**SA type**								26.985***	.003
Entire property	42.9%	54.2%	58.6%	69.8%	46.8%	35.9%	51.9%		
Private bedroom	49.5%	42.7%	38.6%	27.0%	46.8%	46.2%	42.1%		
Shared bedroom	7.6%	3.1%	2.9%	3.2%	6.4%	17.9%	6.0%		
**Ever been a SA host?**								2.922	.712
Yes	1.9%	0.0%	1.4%	1.6%	0.0%	2.6%	1.2%		
No	98.1%	100%	98.6%	98.4%	100.0%	97.4%	98.8%		
**Household composition**								20.905***	.001
Parents	33.3%	49.0%	50.0%	58.7%	46.8%	71.8%	48.6%		
Spouse (Children)	66.7%	51.0%	50.0%	41.3%	53.2%	28.2%	51.4%		
**Total times used SA**								11.968**	.035
≤3	70.5%	54.2%	67.1%	68.3%	61.7%	82.1%	66.0%		
≥4	29.5%	45.8%	32.9%	31.7%	38.3%	17.9%	34.0%		
**Nights**								14.369	.157
1–2	7.9%	30.2%	34.3%	34.9%	36.2%	56.4%	35.0%		
3–4	56.2%	52.1%	45.7%	49.2%	42.6%	38.5%	49.3%		
≥5	12.4%	17.7%	20.0%	15.9%	21.3%	5.1%	15.7%		
**Year of first used SA**								30.970	.190
Before 2008	1.9%	2.1%	1.4%	1.6%	4.3%	2.6%	2.1%		
2008–2010	5.7%	6.2%	10.0%	0.0%	2.1%	10.3%	5.7%		
2011–2012	6.7%	12.5%	8.6%	1.6%	8.5%	2.6%	7.4%		
2013–2014	17.1%	12.5%	12.9%	14.3%	14.9%	5.1%	13.6%		
2015–2016	25.7%	29.2%	32.9%	33.3%	25.5%	15.4%	27.9%		
2017–2018	42.9%	37.5%	34.3%	49.2%	44.7%	64.1%	43.3%		
**SA platform**								16.260*	.092
Xiaozhu, Tujia and others	37.1%	40.6%	42.9%	33.3%	29.8%	46.2%	38.3%		
Airbnb	40.0%	32.3%	28.6%	44.4%	25.5%	20.5%	33.6%		
C-trip	22.9%	27.1%	28.6%	22.2%	44.7%	33.3%	28.1%		

*p<0.1,

**p<0.05,

***p<0.01

The discriminant analysis results comprise one and five discriminant functions for pre-adopters and post-adopters respectively (See Table 5), which are in combination with different segments. In addition, 97.4% of the original cases are correctly classified as pre-adopters and 88.8% are correctly classified as post-adopters, which is regarded as relatively strong support for the cluster analysis results.

**Table 5:** The overall discriminant analysis results.

	Function	Eigenvalue	% of variance	Cumulative %	Canonical Correlation	After Function	Wilks’ Lambda	Chi-square	df	sig.
Pre-adopters	1	2.582	69.6	69.6	0.849	0	0.131	825.72	44	0.000
	2	1.129	30.4	100.0	0.728	1	0.470	307.08	21	0.000
Post-adopters	1	3.526	63.2	63.2	0.883	0	0.046	1254.642	75	0.000
	2	0.996	17.8	81.0	0.706	1	0.210	637.913	56	0.000
	3	0.591	10.6	91.6	0.609	2	0.419	355.681	39	0.000
	4	0.374	6.7	98.3	0.522	3	0.666	166.087	24	0.000
	5	0.093	1.7	100.0	0.292	4	0.915	36.364	11	0.000

^a^97.4% of original cases correctly classified for pre-adopters.

^b^88.8% of original cases correctly classified for post-adopters.

## Discussion, implications, limitations and future research

### Discussion

Pre-adopters tend to be homogeneous, which can be explained by the findings of previous research that pre-adopters differ from post-adopters in terms of prior knowledge in decision-making [19, 36]. Firstly, Pre-adopters’ understanding of the heterogeneity of various types of shared accommodations provided by hosts to travelers is limited. Additionally, the credibility of rooms obtained from social connections could act as crucial information for pre-adopters in booking tourism accommodations, owing to their psychological uncertainty about staying at shared accommodations [19]. Finally, the results can be explained by the unique characteristics of motivations for pre-adopters, who are easily attracted by economic and social connection benefits [5, 28]. Thus, conducting the market segmentation becomes less useful than offering a lower price and word-of-mouth recommendations from their social connections.

There is still no explicit analysis offered on the issue of post-adopters’ willingness to afford extra expenses for some additional service they prefer or simply seek lower prices. The present findings reveal that post-adopters who have used this unique tourism accommodation usually have a stronger perception of its values in both functional and experiential aspects. This is because they tend to hear about more innovative information and realize greater shared accommodation values, service performance and so forth [37]. In other words, motivations for using shared properties are much clearer and in more detail, which echoes six clusters derived from different aspects of post-adopters’ preferences. Therefore, cheaper price is a valid strategy for money-savers, while for the rest five clusters, their willingness to afford extra expenses for some additional service is stronger.

The current study, therefore, explores the motivation evolution process from pre-adopters to post-adopters, which is vital to understanding how market segments change from three to six. Regarding price, shared accommodations are considered a cost-effective alternative to traditional accommodation options [38]. Pre-adopters tend to use social connection information sources to make booking decisions, offering more convenience to book rooms with greater credibility in providing both practical and experiential benefits. For post-adopters, shared accommodation characteristics could distinguish actual travelers effectively because of their growing level of knowledge. Therefore, their willingness to pay for the shared accommodation up-gradation increases. In this sense, six segments appear in post-adopters, which satisfy their preferences in more aspects. Post-adopters are classified in more detail, which can be employed to reduce competition by separating market segments. Among them, money savers and recommendation travelers are practical seekers; experiential seekers are classified as interaction, ethos and novelty seekers.; travelers who have fewer motivations for repeat usage are considered as well.

### Theoretical implications

This study illustrates the importance of studying pre-adopters. Previous literature has indeed linked individuals at different innovation adoption processes with motivational and profile differences [5, 39], including a number of practical and experiential motivations [5] and different educational backgrounds, social status, degree of uncertainty as well as social networking [39]. This study offers a comprehensive classification of traveler types according to time of adoption, rooted in the Innovation Adoption theory represented by Rogers [14] and therefore divides travelers into pre-adopters and post-adopters. This study mainly focuses on the motivations of pre-adopters (refer to potential travelers) and post-adopters (refer to actual travelers). This study extends the issue that pre-adopters are different from post-adopters in using shared properties because it is still not clear if the findings from tourism and wider fields could be appropriate to the shared accommodation industry. Therefore, an important contribution of this study to the tourism market is emphasizing the motivation and market segments for pre-adopters.

This research provides the first comparative look at travelers’ heterogeneity for shared accommodations at the different adoption stages. Pre-adopters are likely to be more homogeneous with a high level of price-performance, as the factors that distinguished travelers previously become decreasingly relevant. However, in order to target post-adopters properly and reduce market competition, it is crucial to conduct the tourism segmentation among heterogeneous travelers.

This study also explores how the motivations of pre-adopters evolve into motivations of post-adopters in shared accommodations. The research contribution is bringing out a new research direction and method. That is, in the remaining tourism market segmentation studies, it’s necessary to discuss the evolution process from pre-to-post adopters. In this sense, this study provides a brand-new understanding of the motivation evolution process, which is also an innovative methodology for future tourism segmentation studies with similar research questions.

### Practical implications

The study serves to help highlight overall marketing insights, to not only encourage more post-experience travelers to re-engage in the shared accommodation experience, but also to highlight to the pre-experience potential users of shared accommodations as to the other benefits—i.e. enhancing the marketing to broaden the appeal. Specifically, this research offers valuable guidelines for addressing the needs and preferences of specific target groups. The results reveal the importance of differentiated strategies for pre-and-post adopters to meet their motivations, improving their experiences, and thus leading to higher satisfaction levels.

Our findings provide a strong indication of the misalignment between provider expectations and consumer intentions, suggesting that further data-driven insights could increase the matching quality of the platform. We deem such an approach worthwhile because it might create awareness and point to blind spots, resulting in proactive interventions and improvements to make the sharing economy more equitable and enjoyable. Shared accommodations and similar home-sharing services providers are able to propound clear guidance of different accommodation types and appeal to hosts to foster positive experiences and customer satisfaction by framing their listings in a more targeted fashion.

### Limitations

Despite the positive results of this study, limitations still exist: first of all, the respondents are located in Shanghai, so it is necessary to enlarge the sample size in order to have an overall understanding of the entire shared accommodation industry in China. In addition, 22 motivations are provided in this study for travelers to use shared accommodations, while constraints are required to be considered in future studies. Finally, motivations are likely to be changed over time [40]. A better understanding of the changes in motivation-based segmentation for travelers at different adoption phases is important to make effective tourism market strategies.

### Future research

As a follow-up to this research that collected valuable data at the pre-COVID-19 stage, we plan to have a significant look at travelers’ motivation-based segmentation at the post-COVID-19 stage in order to make the updated tourism marketing strategies. Second, the constraints of travelers will be considered carefully. Last, it would be worth collecting pre-adopters’ planned travel expenses and post-adopters’ realized travel expenditures, and analyzing the proportion of shared accommodation expenses on overall travel expenditure.

## Supporting information

S1 Table Questionnaire design. (DOCX)

S2 Table Demographic profiles. (DOCX)

S1 Data Pre-adopters. (XLSX)

S2 Data Post-adopters. (XLSX)

## References

[1] National Information and Sharing Economic Research Centre. (2022). *Development of shared accommodation in China Report*.

[2] So K. K. F., Oh H., & Min S. (2018). Motivations and constraints of Airbnb consumers: Findings from a mixed-methods approach. *Tourism Management*, 67(8), 224–236. doi: 10.1016/j.tourman.2018.01.009

[3] Ert E., Fleischer A., & Magen N. (2016). Trust and reputation in the sharing economy: The role of personal photos in Airbnb. *Tourism Management*, 55, 62–73. doi: 10.1016/j.tourman.2016.01.013

[4] Ministry of Culture and Tourism of China. (2022, April 28). Current situation and market analysis of shared accommodation development in China in 2021. https://3g.163.com/dy/article/H623G93P055360U6.html

[5] Guttentag D., Smith S., Potwarka L., & Havitz M. (2018). Why Tourists Choose Airbnb: A Motivation-Based Segmentation Study. *Journal of Travel Research*, 57(3), 342–359. doi: 10.1177/0047287517696980

[6] Wang D., & Nicolau J. L. (2017). Price determinants of sharing economy-based accommodation rental: A study of listings from 33 cities on Airbnb. com. *International Journal of Hospitality Management*, 62, 120–131. doi: 10.1016/j.ijhm.2016.12.007

[7] Guttentag D. (2016). *Why tourists choose Airbnb*: *a motivation-based segmentation study underpinned by innovation concepts*. University of Waterloo, Waterloo, ON. http://hdl.handle.net/10012/10684

[8] Wen J., & Huang S. (2019). Chinese tourists visiting volatile destinations: Integrating cultural values into motivation-based segmentation. *Journal of China Tourism Research*, 15(4), 520–540. doi: 10.1080/19388160.2019.1589610

[9] Quer D., & Peng J. (2022). *Chinese outbound tourism segmentation*: *A systematic review and research agenda*. *Journal of China Tourism Research*, 18(4), 778–808.

[10] Khoo-Lattimore C., & Prayag G. (2015). The girlfriend getaway market: Segmenting accommodation and service preferences. *International Journal of Hospitality Management*, 45, 99–108. doi: 10.1016/j.ijhm.2014.12.003

[11] Lutz C., & Newlands G. (2018). Consumer segmentation within the sharing economy: The case of Airbnb. *Journal of Business Research*, 88(7), 187–196. doi: 10.1016/j.jbusres.2018.03.019

[12] Pike S., Kotsi F., Mathmann F., & Wang D. (2021). Making the right stopover destination choice: The effect of assessment orientation on attitudinal stopover destination loyalty. *Journal of Hospitality and Tourism Management*, 47(2), 462–467. doi: 10.1016/j.jhtm.2021.04.008

[13] Schumpeter J.A. (2007) Theorie der wirtschaftlichen Entwicklung. The Theory of Economic Development. *An inquiry into profits*, *capital*, *credit*, *interest*, *and the business cycle*. New Brunswick, NJ: Transaction Publishers.

[14] Rogers E. M. (2003). *Diffusion of Innovations* (5th ed.). Free Press.

[15] Guttentag D., & Smith S. L. J. (2020). The diffusion of Airbnb: a comparative look at earlier adopters, later adopters, and non-adopters. *Current Issues in Tourism*, 6(1), 1–20. doi: 10.1080/13683500.2020.1782855

[16] Yu, Y. (2021). *Factors affecting young Chinese tourists’ use of Airbnb and the impact of personal values*: *A motivation-based segmentation study* [Doctoral dissertation, University of Otago]. Dunedin, New Zealand. http://hdl.handle.net/10523/10888

[17] De Larrea G. L., Altin M., Koseoglu M. A., & Okumus F. (2021). An integrative systematic review of innovation research in hospitality and tourism. *Tourism Management Perspectives*, 37, 100789. doi: 10.1016/j.tmp.2021.100789

[18] Dabija D. C., Csorba L. M., Isac F. L., & Rusu S. (2022). Building Trust toward Sharing Economy Platforms beyond the COVID-19 Pandemic. *Electronics*, 11(18), 2916. doi: 10.3390/electronics11182916

[19] Jun S. H. (2020). The effects of perceived risk, brand credibility and past experience on purchase intention in the Airbnb context. *Sustainability*, 12(12), 5212. doi: 10.3390/su12125212

[20] Maghrifani D., Liu F., & Sneddon J. (2022). Understanding potential and repeat visitors’ travel intentions: the roles of travel motivations, destination image, and visitor image congruity. *Journal of Travel Research*, 61(5), 1121–1137. doi: 10.1177/00472875211018508

[21] Tussyadiah I. P., & Pesonen J. (2018). Drivers and barriers of peer-to-peer accommodation stay—an exploratory study with American and Finnish travelers. *Current Issues in Tourism*, 21(6), 703–720. doi: 10.1080/13683500.2016.1141180

[22] Huang S., Zhang J., Wang L., & Hua X. (2015). Social friend recommendation based on multiple network correlation. *IEEE Transactions on Multimedia*, 18(2), 287–299. doi: 10.1109/TMM.2015.2510333

[23] Hamari J., Sjöklint M., & Ukkonen A. (2016). The sharing economy: Why people participate in collaborative consumption. *Journal of the Association for Information Science and Technology*, 67(9), 2047–2059. doi: 10.1002/asi.23552

[24] Adner R. (2002). When are technologies disruptive? A demand-based view of the emergence of competition. *Strategic Management Journal*, 23(8), 667–688. doi: 10.1002/smj.246

[25] Chung K. Y., Oh S. Y., Kim S. S., & Han S. Y. (2004). Three representative market segmentation methodologies for hotel guest room customers. *Tourism Management*, 25(4), 429–441. doi: 10.1016/S0261-5177(03)00115-8

[26] Khoo-Lattimore C., Prayag G., & Disegna M. (2019). Me, My Girls, and the Ideal Hotel: Segmenting Motivations of the Girlfriend Getaway Market Using Fuzzy C-Medoids for Fuzzy Data. *Journal of Travel Research*, 58(5), 774–792. doi: 10.1177/0047287518778154

[27] Sparks B., & Pan G. W. (2009). Chinese outbound tourists: Understanding their attitudes, constraints and use of information sources. *Tourism Management*, 30(4), 483–494. doi: 10.1016/j.tourman.2008.10.014

[28] Tran T. H., & Filimonau V. (2020). The (de) motivation factors in choosing Airbnb amongst Vietnamese consumers. *Journal of Hospitality and Tourism Management*, 42, 130–140. doi: 10.1016/j.jhtm.2019.10.011

[29] Wu J., Zeng M., & Xie K. L. (2017). Chinese travelers’ behavioral intentions toward room-sharing platforms: The influence of motivations, perceived trust, and past experience. *International Journal of Contemporary Hospitality Management*, 29(10), 2688–2707. doi: 10.1108/IJCHM-08-2016-0481

[30] Dolnicar S. (2020). Market segmentation for e-Tourism. *Handbook of e-Tourism*, 1–15. doi: 10.1007/978-3-030-05324-6_53-1

[31] Thompson S. K. (2012). *Sampling*. John Wiley & Sons.

[32] Mu-niao short rentals. (2023). *2023 May Day Holiday Shared accommodation Consumption Report*.

[33] Sarstedt M., & Mooi E. (2014). The market research process. In *A Concise Guide to Market Research*. Springer.

[34] Vejlgaard H. (2018). Process knowledge in the innovation-decision period. In *Digital Communication Management*. IntechOpen..

[35] Nezakati H. (2013). Globalization and Consumer Behavior: Global Marketing Strategies Implication-Homogeneity and Heterogeneity (Preliminary Study). *Journal of Social & Development Sciences*, 4(1), 1–5. doi: 10.22610/jsds.v4i1.729

[36] Sharifpour M., Walters G., Ritchie B. W., & Winter C. (2014). Investigating the role of prior knowledge in tourist decision making: A structural equation model of risk perceptions and information search. *Journal of Travel Research*, 53(3), 307–322. doi: 10.1177/0047287513500390

[37] Young H. P. (2009). Innovation Diffusion in Heterogeneous Populations. *Social Science Electronic Publishing*, 99(5), 1899–1924. doi: http%3A//dx.doi.org/10.2139/ssrn.1024811

[38] Zhang C. X., Isaeva N., & Li S. (2022). When hosts trust guests and sharing platforms: trust in sharing economy. *Journal of China Tourism Research*, 18(3), 630–650. doi: 10.1080/19388160.2021.1952124

[39] Dedehayir O., Ortt R. J., Riverola C., & Miralles F. (2017). Innovators and early adopters in the diffusion of innovations: A literature review. *International Journal of Innovation Management*, 21(8), 1–27. doi: 10.1142/S1363919617400102

[40] Ryan R. M., & Deci E. L. (2017). *Self-Determination Theory*: *Basic psychological needs in motivation*, *development*, *and wellness*. Guilford Publications.

