# Supplementary material for: Consumer heterogeneity for shared accommodations at pre-and-post adoption stages: Insights from travelers in Shanghai, China
Source: PLoS One. 2023 Jun 23;18(6):e0286868. doi: 10.1371/journal.pone.0286868 (PMC10289423; doi:10.1371/journal.pone.0286868)
Supplement: S1 Table — (DOCX) [file pone.0286868.s001.docx]

**S1 Table. Questionnaire design.**

| Likert questions | Literature |
| --- | --- |
| The cheaper price is an important factor for me to select shared accommodations. | [1] |
| A more convenient location is an important factor for me to select shared accommodations. | [2] |
| A larger space is an important factor for me to select shared accommodations. | [3] |
| Better household facilities are an important factor for me to select shared accommodations. | [3] |
| I am interested in shared accommodations because others want to live in them. | [4] |
| I am interested in shared accommodations because others recommend them to me. | [4] |
| I am interested in shared accommodations because I can get useful travel tips and information from my host. | [2] |
| I want to book shared accommodations because of my travel party size. | [2] |
| I am interested in shared accommodations because of their additional services. | [4] |
| I am interested in shared accommodations because I can share my experiences with my friends or relatives. | [2] |
| I am interested in shared accommodations because I want to have a nonstandard experience. | [5] |
| I am interested in shared accommodations because I want to live in a non-touristy neighborhood. | [2] |
| I am interested in shared accommodations because I think I can have more choices of accommodations in the future. | [5] |
| I am interested in shared accommodations because I want to interact with the host and the locals. | [2] |
| I am interested in shared accommodations because they can give me a kind of home feeling. | [2] |
| I am interested in shared accommodations because I want to try a new and different accommodation mode. | [5] |
| I am interested in shared accommodations because it is environmentally friendly. | [6] |
| I am interested in shared accommodations because I like its philosophy. | [6] |
| I am interested in shared accommodations because I want to have an authentic local experience. | [2] |
| I am interested in shared accommodations because it’s fun. | [7] |
| I am interested in shared accommodations because I enjoy staying in them. | [7] |
| I am interested in shared accommodations for the pleasure of discovering new accommodation types. | [7] |

**References**

1. Adner, R. (2002). When are technologies disruptive? A demand-based view of the emergence of competition.

*Strategic Management Journal, 23*(8), 667-688. <https://doi.org/10.1002/smj.246>

2. Guttentag, D., Smith, S., Potwarka, L., & Havitz, M. (2018). Why Tourists Choose Airbnb: A Motivation-Based Segmentation Study. *Journal of Travel Research, 57*(3), 342-359. https://doi.org/10.1177/0047287517696980

3. Quinby, D., & Gasdia, M. (2014). Share this! Private accommodation and the rise of the new gen renters. *Report. PhoCusWright*.

https://doi.org/10.1080/19388160.2021.1966563

4. Mohlmann, M. (2015). Collaborative consumption: determinants of satisfaction and the likelihood of using a sharing economy option again. *Journal of Consumer Behaviour, 14*(3), 193-207. https://doi.org/10.1002/cb.1512

5. Ert, E., Fleischer, A., & Magen, N. (2016). Trust and reputation in the sharing economy: The role of personal photos in Airbnb. *Tourism Management, 55*, 62-73. <https://doi.org/10.1016/j.tourman.2016.01.013>

6. Chase, R. (2015) *Peers Inc: How people and platforms are inventing the collaborative economy and reinventing capitalism*. PublicAffairs.

7. So, K. K. F., Oh, H., & Min, S. (2018). Motivations and constraints of Airbnb consumers: Findings from a mixed-methods approach. *Tourism Management, 67*(8), 224-236. https://doi.org/10.1016/j.tourman.2018.01.009
