# Supplementary material for: Consumer heterogeneity for shared accommodations at pre-and-post adoption stages: Insights from travelers in Shanghai, China
Source: PLoS One. 2023 Jun 23;18(6):e0286868. doi: 10.1371/journal.pone.0286868 (PMC10289423; doi:10.1371/journal.pone.0286868)
Supplement: S2 Table — (DOCX) [file pone.0286868.s002.docx]

**S2 Table. Demographic profiles.**

|  | Pre-adopters | | Post-adopters | |
| --- | --- | --- | --- | --- |
| Characteristics | n | % | n | % |
| **Gender** |  |  |  |  |
| Male | 145 | 34.50% | 139 | 33.10% |
| Female | 275 | 65.50% | 281 | 66.90% |
| **Age** |  |  |  |  |
| 18-20 | 60 | 14.30% | 56 | 13.30% |
| 21-30 | 197 | 46.90% | 211 | 50.20% |
| 31-40 | 116 | 27.60% | 123 | 29.30% |
| 41-50 | 34 | 8.10% | 25 | 6.00% |
| 51-60 | 10 | 2.40% | 5 | 1.20% |
| ≥61 | 3 | 0.70% | 0 | 0% |
| **Job** |  |  |  |  |
| Civil servant | 47 | 11.20% | 49 | 11.70% |
| Worker | 20 | 4.80% | 3 | 0.70% |
| Company staff | 146 | 34.80% | 183 | 43.60% |
| Service industry | 44 | 10.50% | 30 | 7.10% |
| Financial industry | 28 | 6.70% | 35 | 8.30% |
| Farmer | 2 | 0.50% | 0 | 0% |
| Student | 93 | 22.10% | 103 | 24.50% |
| Freelancers | 30 | 7.10% | 10 | 2.40% |
| Other | 10 | 2.40% | 7 | 1.70% |
| **Education** |  |  |  |  |
| Senior high school and below | 53 | 12.60% | 21 | 5.00% |
| College | 108 | 25.70% | 72 | 17.10% |
| Bachelor | 224 | 53.30% | 274 | 65.20% |
| Master and above | 35 | 8.30% | 53 | 12.60% |
| **Travel composition (Last time)** |  |  |  |  |
| Alone | 21 | 5.00% | 13 | 3.10% |
| Friends | 210 | 50.00% | 54 | 12.90% |
| Relatives | 46 | 11.00% | 110 | 26.20% |
| Spouse | 109 | 26.00% | 15 | 3.60% |
| Children | 29 | 6.90% | 5 | 1.20% |
| Other | 5 | 1.20% | 0 | 0.00% |
| **Stay (Last time)** |  |  |  |  |
| 1 night |  |  | 32 | 7.60% |
| 2 nights |  |  | 115 | 27.40% |
| 3 nights |  |  | 163 | 38.80% |
| 4 nights |  |  | 44 | 10.50% |
| 5 nights |  |  | 46 | 11.00% |
| 6 nights |  |  | 5 | 1.20% |
| Over 6 nights |  |  | 15 | 3.60% |
| **Booking platform (Last time)** |  |  |  |  |
| Xiaozhu short rentals |  |  | 29 | 6.90% |
| Airbnb |  |  | 141 | 33.60% |
| Tujia.com |  |  | 23 | 5.50% |
| Zhenguo |  |  | 14 | 3.30% |
| C-trip |  |  | 118 | 28.10% |
| Meituan |  |  | 44 | 10.50% |
| Qunar.com |  |  | 33 | 7.90% |
| Other |  |  | 18 | 4.30% |
